# Postoperative delirium risk in elderly high-risk patients undergoing hip and knee arthroplasty (ASA Ⅲ-Ⅳ): construction and validation of a nomogram model based on frail frailty index

**DOI:** 10.3389/fmed.2026.1860359

**Published:** 2026-09-04

**Authors:** Shaobo Ma, Suoping Yang

**Affiliations:** 1 Department of Anesthesiology, Yinchuan Guolong Orthopedic Hospital, Yinchuan, China; 2 Department of Orthopedics, Xianyang Hospital of Yan'an University, Xianyang, China

**Keywords:** FRAIL frailty index, hip and knee arthroplasty, nomogram, postoperative delirium, prediction model

## Abstract

**Objective:**

To create and verify a nomogram for predicting postoperative delirium (POD) in elderly patients having knee or hip replacements based on the FRAIL frailty index.

**Methods:**

582 elderly patients with American Society of Anesthesiologists (ASA) grades III-IV who had elective primary total hip or knee arthroplasty at Yinchuan Guolong Orthopedic Hospital between January 2022 and June 2025 were the subject of a retrospective study. The patients were randomly assigned to a training set (*n* = 407) and a validation set (*n* = 175) at a 7:3 ratio. Least absolute shrinkage and selection operator regression was used to screen candidate variables, followed by multivariable logistic regression to identify independent risk factors for POD. A nomogram was constructed based on these factors. Area under the receiver operating characteristic curve, calibration curves, and decision curve analysis (DCA) were used to assess model discrimination, calibration, and clinical utility in both cohorts. SHapley Additive exPlanations (SHAP) analysis was used to improve model interpretability.

**Results:**

Preoperative serum albumin (OR = 0.901, 95% CI: 0.830–0.978), age (OR = 1.092, 95% CI: 1.023–1.166), preoperative frailty index (OR = 2.021, 95% CI: 1.612–2.534), ASA grade IV (OR = 2.105, 95% CI: 1.131–3.917), preoperative Chinese Mini Mental Status <27 (OR = 3.608, 95% CI: 1.878–6.931), and early postoperative Numerical Rating Scale (NRS) score ≥6 (OR = 5.998, 95% CI: 3.028–11.880) emerged as six independent risk factors for POD. With AUCs of 0.864 (95% CI: 0.819–0.909) in the training set and 0.875 (95% CI: 0.800–0.949) in the validation set, the nomogram demonstrated good discrimination. Additionally, calibration curves demonstrated good agreement with Brier scores of 0.107 and 0.094, respectively. Clinical net benefit was verified by DCA over a broad range of threshold probabilities (8%–45%). With directional effects in line with clinical knowledge, SHAP analysis identified the frailty index as the most significant predictor, followed by postoperative NRS and preoperative albumin.

**Conclusion:**

The nomogram, which is based on six clinical factors, successfully predicts POD risk in elderly arthroplasty patients, exhibiting good discrimination, calibration, and utility for early risk identification and customized prevention.

## Introduction

The gold standard for treating end-stage joint conditions such severe osteoarthritis and rheumatoid arthritis is hip and knee joint replacement surgery, which can greatly enhance an aged patient's quality of life and joint function (1, 2). However, the number of elderly patients undergoing hip and knee arthroplasty has been rising annually due to the aging population, accompanied by shifts in case-mix over time (3, 4). Postoperative delirium (POD) is a common acute neurocognitive problem consequence that affects older patients during the postoperative period. It typically manifests one to seven days following surgery and is characterized by attention fluctuations, awareness difficulties, and cognitive dysfunction (5). According to studies, the incidence of POD is 15% to 35% in older, high-risk patients having hip and knee joint replacement surgery, which is much greater than the general population (6, 7). Additionally, POD can result in longer hospital admissions, higher medical expenses, higher rates of surgical complications and mortality, and permanent cognitive deterioration in some patients, which can have a major impact on the prognosis of rehabilitation (8).

The pathophysiology of POD is yet unclear. Many risk factors during the postoperative period, accumulated underlying disorders, surgical trauma stress, and degenerative changes in brain function in senior patients are usually thought to be associated with it (9). ASA grade III-IV patients have severe systemic diseases, such as severe cardiovascular diseases and respiratory insufficiency, and their physiological reserve capacity is significantly reduced, resulting in poor tolerance to surgical anesthesia and further increasing the risk of POD (10).

Adverse surgical outcomes in older persons are closely correlated with frailty, a multifaceted condition that reflects the decrease of physiological reserve across numerous systems (11). The International Society of Nutrition and Aging developed the FRAIL scale in 2008 as a quick screening tool spanning five domains: fatigue, resistance, ambulation, sickness, and weight loss (12). The notion of frailty was initially presented in geriatric medicine in the 1970s (13). In high-risk populations, frailty may synergistically interact with ASA classification to determine patients' tolerance to surgical stress and the subsequent risk of POD. Physiological vulnerability can be quantified using the FRAIL scale, a straightforward and validated method for evaluating frailty. Preoperative fragility has been shown to be an independent risk factor for POD in patients undergoing hip or knee replacement, with a significant correlation found in a large cohort research (14). In China, the FRAIL scale has also been validated and applied in older surgical populations. Preoperative fragility was found to be an independent risk factor for postoperative delirium in a study of elderly individuals receiving spine surgery (15). However, the role of frailty as a continuous predictor in POD risk stratification, specifically in ASA grade III–IV older adults having hip and knee arthroplasty, remains underexplored.

Although some studies have constructed POD prediction models for older adults undergoing hip and knee arthroplasty, the quality and generalizability of these models, particularly for high-risk subgroups such as ASA grade III-IV patients, remain inadequately validated (16). The nomogram, as a visual prediction tool, can convert complex regression equations into intuitive graphics, facilitating clinical medical staff to quickly calculate individual risk probabilities and has good clinical practicability. With an emphasis on the FRAIL-based frailty score, this study sought to evaluate independent risk variables for POD in ASA grade III–IV elderly high-risk patients undergoing hip and knee arthroplasty. A targeted nomogram prediction model incorporating frailty was then constructed and validated, providing a new and effective tool for precise POD prevention and control in clinical practice.

## Materials and methods

### Study design and participants

**Figure 1:**
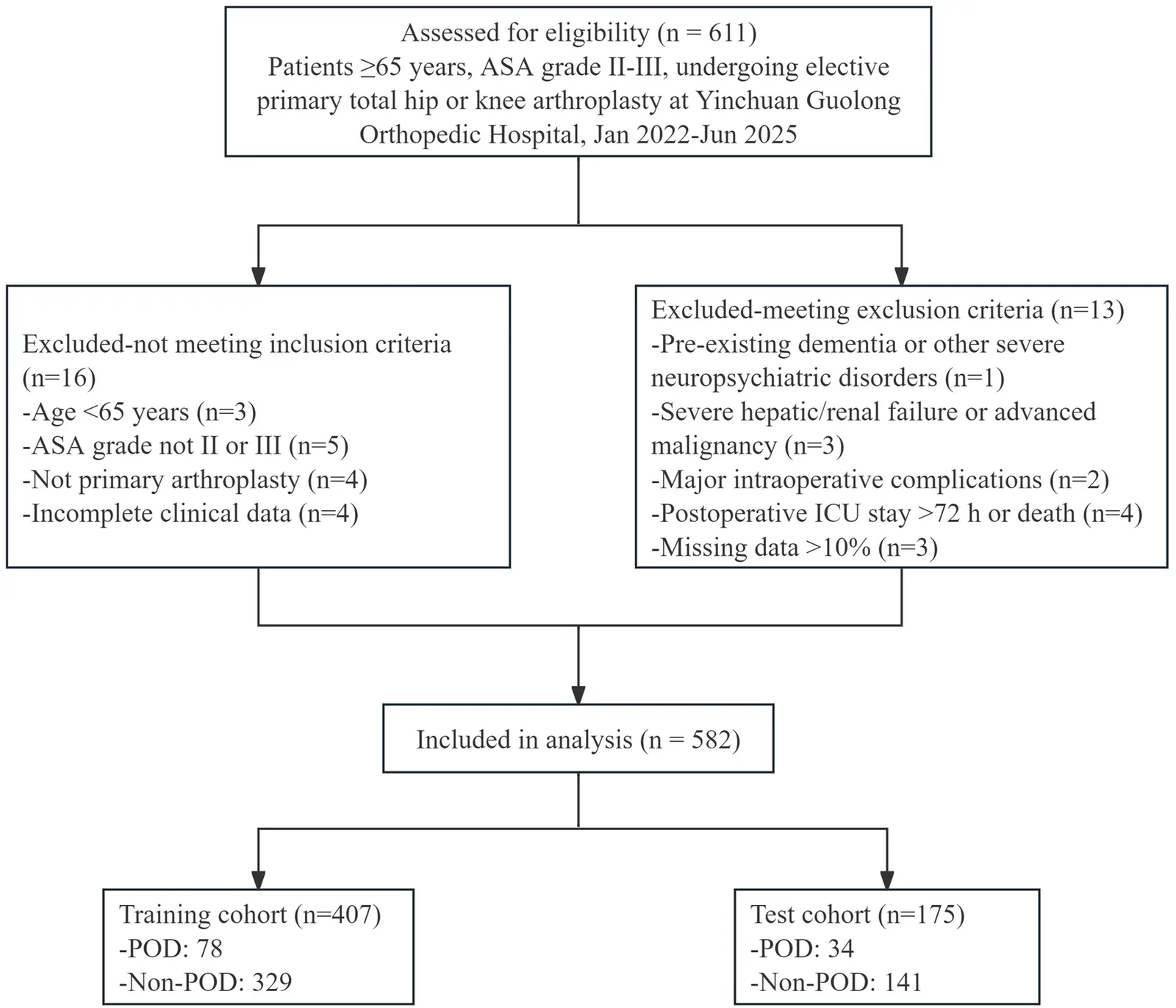
Flowchart of patient selection for the study cohort. POD indicates Postoperative delirium; ASA, American Society of Anesthesiologists.

In the Orthopedics Department of Yinchuan Guolong Orthopedic Hospital, 611 senior patients who underwent elective primary hip and knee joint replacement surgery between January 2022 and June 2025 had their clinical data retrospectively collected. Criteria for inclusion: ① 65 years of age or older; ② ASA grade III-IV (17); ③Primary total hip arthroplasty or total knee replacement; ④ Complete clinical data. Exclusion criteria: ① Preoperative diagnosis of dementia (18), schizophrenia, etc. mental neurological diseases; ② Severe liver and kidney failure including Child-Pugh C grade or chronic kidney disease stage 5, or advanced malignant tumors; ③ Severe complications during surgery such as cardiac arrest, massive bleeding (bleeding volume >1,000 mL); ④ Postoperative stay in ICU for more than 72 h or direct death; ⑤ Missing rate of clinical data >10%. Finally, 582 patients were included in the study, among which 16 did not meet the inclusion criteria and 13 met the exclusion criteria. The patients were split into a training set and an internal validation set at a ratio of 7:3 using the random number table approach. The training set, which included 329 patients without postoperative delirium and 78 with it, was utilized to build a nomogram model and screen independent prognostic features. The predictive efficacy of the model was validated using an internal validation set of 175 patients, 34 of whom had postoperative delirium and 141 of whom did not (Figure 1). The Yinchuan Guolong Orthopedic Hospital's Medical Ethics Committee gave their approval for this study [Ethical Approval Number: (2026)05]. Patients' informed agreement was not required because the study was retrospective. The Helsinki Declaration's tenets were closely followed during the study procedure.

### Diagnostic criteria and outcome indicators

The Confusion Assessment Method (CAM) is combined with the Diagnostic and Statistical Manual of Mental Disorders-Fifth Edition (DSM-5) (19) criteria to diagnose POD: ① Acute onset with variable conditions; ② Attention disorder; ③ Thought disarray; ④ Change in consciousness level. If any three of the above are met, POD can be diagnosed. Two neurology physicians who have received unified training independently review the patient's medical records, including nursing records, course of treatment records, and psychiatric consultation opinions, and extract keywords such as delirium, agitation, and disorientation. When there is a diagnostic disagreement, the final diagnosis is determined through double-checking and consultation with a third party. The outcome indicator is whether POD occurs within 7 days after surgery.

### Observation indicators

The hospital information system was used to retroactively retrieve all of the study's data. Based on the standardized data collection form, two researchers with unified training independently collected the following data from the laboratory information system, the nursing evaluation system, and the electronic medical record system:

Demographic characteristics: Collect the patient's age, sex, body mass index (BMI), educational level, mode of living, smoking history, and drinking history.

Preoperative comorbidities and laboratory indicators: Collect the patient's ASA classification (grade III/IV), underlying diseases, preoperative serum albumin (ALB), preoperative anemia, and preoperative sleep disorders. The ASA classification is based on the American Society of Anesthesiologists classification criteria (17): Grade III indicates that the patient has severe systemic diseases with limited function but no decompensation; Grade IV indicates that the patient has a life-threatening severe systemic disease. Based on prior clear diagnosis records, underlying disorders include diabetes, hypertension, coronary heart disease, stroke, chronic heart failure (CHF), and chronic obstructive pulmonary disease (COPD). Hemoglobin levels below 110 g/L are considered preoperative anemia (20). Preoperative sleep disorders are defined as positive if described in the medical record as insomnia, difficulty falling asleep, easily waking up, or if there is a clear diagnosis of sleep disorder.

Preoperative cognitive function: The Chinese Mini Mental Status (CMMS) (21) was used to assess the patients' preoperative cognitive function. With a total score ranging from 0 to 30, this measure comprises five dimensions: orientation (10 points), memory (3 points), attention and calculation (5 points), recall (3 points), and language (9 points). Individuals who scored less than 17 points for illiterates, less than 20 points for primary school graduates, and less than 24 points for those with secondary education or more were considered to have dementia and were excluded based on their educational level (18). Cognitive dysfunction was defined as a score of less than 27 points on the CMMS, which was reported for the study's subjects.

Preoperative frailty status: The patients' preoperative frailty state was evaluated using the validated Chinese version of the FRAIL scale (22). Relevant baseline information was routinely collected by anesthesiology nurses during preoperative anesthesia assessment one day before surgery, and retrospectively abstracted from electronic medical records in this study. This scale has five dimensions, and each dimension that satisfies the definition is worth one point. The total score is between 0 and 5. The specific scoring criteria are as follows: ① Weight loss: Weight loss of ≥5% in the past 1 year without intentional weight loss, or significant weight loss reported by the patient; ② Fatigue: Feeling fatigued for most of the past 4 weeks; ③ Resistance walking: Can independently ascend one flight of stairs? If unable to complete or requires assistance, it is recorded as positive; ④ Walking ability: Can independently walk one block, approximately 200–300 meters. If unable to complete or requiring assistance, it is recorded as positive.⑤ Number of diseases: If there are more than five chronic illnesses, including diabetes, hypertension, coronary heart disease, stroke, chronic heart failure, chronic obstructive pulmonary disease, arthritis, and renal disease. Wu et al. (23) reported that the FRAIL scale may accurately predict the likelihood of short-term problems, such as postoperative delirium, in elderly patients following primary total knee arthroplasty.

Perioperative related indicators: Patient information was collected, including the type of surgery, the length of the procedure, the anesthesia technique, the amount of blood lost during the procedure, the postoperative transfer to the intensive care unit (ICU), postoperative hypoxemia, postoperative C-reactive protein (CRP), the early postoperative pain score, and the use of sedatives and analgesics following the procedure. Postoperative hypoxemia is defined as arterial blood oxygen saturation <90% for ≥10 min within 24 h after surgery (24). The Numerical Rating Scale (NRS) (25) is used for assessment. The NRS score is evaluated on a scale of 0–10 to assess the severity of pain, with higher scores indicating more severe pain. Postoperative pain was assessed using the NRS at two time points during the post-anesthesia care unit stay, upon arrival when the patient was alert and able to respond to verbal commands, and 30 min thereafter. The higher of the two scores was recorded as the early postoperative NRS score and used for analysis. Mild pain is represented by a score of ≤5, moderate pain by a score of 6–7, and severe pain by a score of ≥8 (26). Therefore, in this study, the NRS score is dichotomized at 6: <6 is classified as mild pain, ≥6 as moderate/severe pain. The use of postoperative sedative and analgesic drugs refers to whether pethidine was administered (27).

### Quality control

To ensure data accuracy and consistency, all researchers underwent unified training prior to the study. Two data collectors independently extracted and cross-checked the information, while two neurologists independently diagnosed POD using DSM-5 and CAM criteria, with a Kappa value >0.8 indicating good consistency. Data were double-entered and logically verified, and any ambiguous records were clarified through telephone follow-up.

### Statistical analysis

SPSS 27.0 (IBM Corp., Armonk, NY, USA) was used for data analysis. Firstly, the data were cleaned by removing duplicate records. For the cases included in the analysis, if there were missing values for a certain variable and the missing rate was less than 10%, the multiple imputation method was used for processing; if the cases had missing values in key variables that prevented imputation, they were excluded. The Shapiro–Wilk test was used to determine whether measurement data was normal. Measurement data with a normal distribution were expressed as the mean ± standard deviation (SD), and the independent sample *t*-test was used to compare the groups; measurement data without a normal distribution were expressed as the median (quartiles), and the Wilcoxon rank sum test was used to compare the groups. Frequency (n) and percentage (%) were used to express categorical data, and Fisher's exact probability method or the *χ*² test were used to compare groups. All candidate variables were screened using least absolute shrinkage and selection operator (LASSO) regression (10-fold cross-validation), and the non-zero coefficient variables at the minimal penalty coefficient *λ* were chosen as possible risk factors. The odds ratio (OR) and 95% confidence interval (CI) were computed to identify the independent risk factors of POD after the variables chosen by LASSO were incorporated into the multivariate logistic regression analysis. A nomogram prediction model was built using the independent risk factors chosen by the multivariate Logistic regression analysis. Internal validation was performed using bootstrap with 1,000 resamples. The model's discrimination was assessed using the area under the receiver operating characteristic (ROC) curve; an area under the curve (AUC) > 0.8 denotes good discrimination. The model's calibration was assessed using the calibration curve and the Hosmer-Lemeshow goodness-of-fit test, with *P* > 0.05 indicating satisfactory model calibration; the model's clinical value was assessed using decision curve analysis (DCA). Python 3.10 was used to perform SHapley Additive exPlanations (SHAP) analysis, which allowed for the quantification of each predictor's contribution to the model output, the visualization of feature importance, and the interpretation of the direction and amount of variable effects on both individual and overall predictions. Statistical significance was defined as a two-sided *P* < 0.05.

## Results

### Comparison of baseline characteristics between the training and test cohorts

A total of 582 patients met the inclusion criteria. Among them, 407 cases were assigned to the training cohort, including 214 cases of total hip arthroplasty and 193 cases of total knee arthroplasty, with 78 cases of POD yielding an incidence rate of 19.17%. The validation cohort comprised 175 cases, including 89 cases of total hip arthroplasty and 86 cases of total knee arthroplasty, with 34 cases of POD yielding an incidence rate of 19.43%. There were no statistically significant differences between the two groups in terms of demographics, preoperative comorbidities, and perioperative indicators (all *P* > 0.05) (Table 1).

**Table 1:** Comparison of baseline characteristics between the training and internal test cohorts.

Characteristics	Training cohort (*n* = 407)	Validation cohort (*n* = 175)	Statistic	*P*
Age (years, mean ± SD)	76.66 ± 5.95	76.32 ± 5.51	0.637^a^	0.525
Sex (*n*, %)			0.923^b^	0.337
Male	185 (45.45)	72 (41.14)		
Female	222 (54.55)	103 (58.86)		
BMI (kg/ m², mean ± SD)	24.03 ± 3.34	24.21 ± 2.78	−0.671^a^	0.502
Educational level (*n*, %)			1.048^b^	0.592
Illiterate	68 (16.71)	27 (15.43)		
Primary school	156 (38.33)	75 (42.86)		
Junior high school and above	183 (44.96)	73 (41.71)		
Mode of living (*n*, %)			0.629^b^	0.428
Live alone	240 (58.97)	97 (55.43)		
Live with family	167 (41.03)	78 (44.57)		
Smoking history (*n*, %)	67 (16.46)	30 (17.14)	0.041^b^	0.840
Drinking history (*n*, %)	95 (23.34)	36 (20.57)	0.538^b^	0.463
ASA Classification (*n*, %)			0.078^b^	0.780
Grade Ⅲ	397 (97.54)	170 (97.14)		
Grade Ⅳ	10 (2.46)	5 (2.86)		
Combined hypertension (*n*, %)	183 (44.96)	82 (46.86)	0.177^b^	0.674
Combined diabetes (*n*, %)	100 (24.57)	38 (21.71)	0.552^b^	0.458
Combined hyperlipidemia (*n*, %)	42 (10.32)	12 (6.86)	1.743^b^	0.187
Combined CHF (*n*, %)	44 (10.81)	29 (16.57)	3.702^b^	0.054
Combined Stroke (*n*, %)	62 (15.23)	27 (15.43)	0.004^b^	0.952
Combined COPD (*n*, %)	32 (7.86)	16 (9.14)	0.265^b^	0.607
Preoperative serum ALB (g/L, mean ± SD)	34.20 ± 3.82	34.25 ± 3.33	−0.165^a^	0.869
Preoperative anemia (*n*, %)	81 (19.90)	29 (16.57)	0.885^b^	0.347
Preoperative sleep disorders (*n*, %)	131 (32.19)	52 (29.71)	0.347^b^	0.556
Preoperative CMMS score (*n*, %)			2.244^b^	0.134
≥27	297 (72.97)	138 (78.86)		
<27	110 (27.03)	37 (21.14)		
Preoperative Frail frailty index (*n*, %)			−0.933^c^	0.351
Surgical type (*n*, %)			0.146^b^	0.703
Total hip arthroplasty	214 (52.58)	89 (50.86)		
Total knee arthroplasty	193 (47.42)	86 (49.14)		
Operation duration [min, M (Q_1_，Q_3_)]	128.00 (101.00, 156.00)	129.00 (112.00, 140.00)	−0.476^c^	0.634
Anesthesia method (*n*, %)			0.175^b^	0.676
General anesthesia	217 (53.32)	90 (51.43)		
Neuraxial anesthesia	190 (46.68)	85 (48.57)		
Intraoperative blood loss volume [mL, M (Q_1_，Q_3_)]	162.00 (130.00, 190.00)	156.00 (139.00, 174.00)	−1.539^c^	0.124
ICU admission (*n*, %)	28 (6.88)	19 (10.86)	2.608^b^	0.106
Postoperative hypoxemia (*n*, %)	63 (15.48)	33 (18.86)	1.014^b^	0.314
Postoperative CRP [mg/L, M (Q_1_，Q_3_)]	21.00 (13.80, 30.00)	23.00 (17.00, 30.60)	−1.950^c^	0.051
Early postoperative NRS score (*n*, %)			0.305^b^	0.581
≥6	105 (25.80)	49 (28.00)		
<6	302 (74.20)	126 (72.00)		
Use pethidine after surgery (*n*, %)	64 (15.72)	30 (17.14)	0.182^b^	0.670

SD, standard deviation; POD, postoperative delirium; BMI, body mass index; ASA, American Society of Anesthesiologists; ALB, albumin; CHF, chronic heart failure; COPD, chronic obstructive pulmonary disease; CMMS, Chinese mini mental status; ICU, intensive care unit; CRP, C-reactive protein; NRS, numerical rating scale.

a independent samples *t*-test.

b Chi-square test.

c Mann–Whitney *U* test.

### Univariate analysis of risk factors for POD

The POD group and the non-POD group differed statistically significantly in terms of age, ASA classification, combined stroke, combined COPD, preoperative serum ALB, preoperative CMMS score, preoperative Frail frailty index, anesthesia method, ICU admission, postoperative hypoxemia, and early postoperative NRS score, according to the single-factor analysis results (all *P* < 0.05). The LASSO regression analysis that followed included these variables (Table 2).

**Table 2:** Univariate analysis of risk factors for POD.

Characteristics	POD group (*n* = 78)	Non-POD group (*n* = 329)	Statistic	*P*
Age (years, mean ± SD)	77.65 ± 4.05	76.16 ± 5.13	2.768^a^	0.006
Sex (*n*, %)			1.322^b^	0.250
Male	40 (51.28)	145 (44.07)		
Female	38 (48.72)	184 (55.93)		
BMI (kg/m², mean ± SD)	24.52 ± 3.23	23.91 ± 3.36	1.443^a^	0.150
Educational level (*n*, %)			5.280^b^	0.071
Illiterate	16 (20.51)	52 (15.81)		
Primary school	36 (46.15)	120 (36.47)		
Junior high school and above	26 (33.33)	157 (47.72)		
Mode of living (*n*, %)			3.216^b^	0.073
Live alone	53 (67.95)	187 (56.84)		
Live with family	25 (32.05)	142 (43.16)		
Smoking history (*n*, %)	14 (17.95)	53 (16.11)	0.155^b^	0.694
Drinking history (*n*, %)	23 (29.49)	72 (21.88)	2.037^b^	0.154
ASA Classification (*n*, %)			-	<0.001
Grade Ⅲ	71 (91.03)	326 (99.09)		
Grade Ⅳ	7 (8.97)	3 (0.91)		
Combined hypertension (*n*, %)	41 (52.56)	142 (43.16)	2.253^b^	0.133
Combined diabetes (*n*, %)	22 (28.21)	78 (23.71)	0.688^b^	0.407
Combined hyperlipidemia (*n*, %)	7 (8.97)	35 (10.64)	0.189^b^	0.664
Combined CHF (*n*, %)	12 (15.38)	32 (9.73)	2.094^b^	0.148
Combined Stroke (*n*, %)	18 (23.08)	44 (13.37)	4.597^b^	0.032
Combined COPD (*n*, %)	11 (14.10)	21 (6.38)	5.187^b^	0.023
Preoperative serum ALB (g/L, mean ± SD)	33.47 ± 3.94	34.89 ± 3.74	−2.987^a^	0.003
Preoperative anemia (*n*, %)	16 (20.51)	65 (19.76)	0.023^b^	0.880
Preoperative sleep disorders (*n*, %)	32 (41.03)	99 (30.09)	3.454^b^	0.063
Preoperative CMMS score (*n*, %)			17.193^b^	<0.001
≥27	43 (55.13)	257 (78.12)		
<27	35 (44.87)	72 (21.88)		
Preoperative Frail frailty index [point, M (Q_1_，Q_3_)]	3.00 (1.00, 4.00)	1.00 (1.00, 2.00)	6.286^c^	<0.001
Surgical type (*n*, %)			0.062^b^	0.803
Total hip arthroplasty	42 (53.85)	172 (52.28)		
Total knee arthroplasty	36 (46.15)	157 (47.72)		
Operation duration [min, M (Q_1_，Q_3_)]	129.00 (99.50, 155.00)	124.50 (106.75, 159.00)	−0.767^c^	0.443
Anesthesia method (*n*, %)			5.646^b^	0.017
General anesthesia	51 (65.38)	166 (50.46)		
Neuraxial anesthesia	27 (34.62)	163 (49.54)		
Intraoperative blood loss volume [mL, M (Q_1_，Q_3_)]	172.00 (132.50, 194.25)	161.00 (129.50, 188.00)	−1.341^c^	0.180
ICU admission (*n*, %)	10 (12.82)	18 (5.47)	5.316^b^	0.021
Postoperative hypoxemia (*n*, %)	21 (26.92)	36 (10.94)	13.370^b^	<0.001
Postoperative CRP [mg/L, M (Q_1_，Q_3_)]	25.95 (14.65, 34.83)	22.50 (15.50, 28.60)	−1.938^c^	0.053
Early postoperative NRS score (*n*, %)			28.000^b^	<0.001
≥6	37 (47.44)	62 (18.84)		
<6	41 (52.56)	267 (81.16)		
Use pethidine after surgery (*n*, %)	17 (21.79)	47 (14.29)	2.683^b^	0.101

SD, standard deviation; POD, postoperative delirium; BMI, body mass index; ASA, American Society of Anesthesiologists; ALB, albumin; CHF, chronic heart failure; COPD, chronic obstructive pulmonary disease; CMMS, Chinese mini mental status; ICU, intensive care unit; CRP, C-reactive protein; NRS, numerical rating scale.

a independent samples t-test.

b Chi-square test.

c Mann–Whitney *U* test.

### LASSO regression for searching potential risk variables

**Figure 2:**
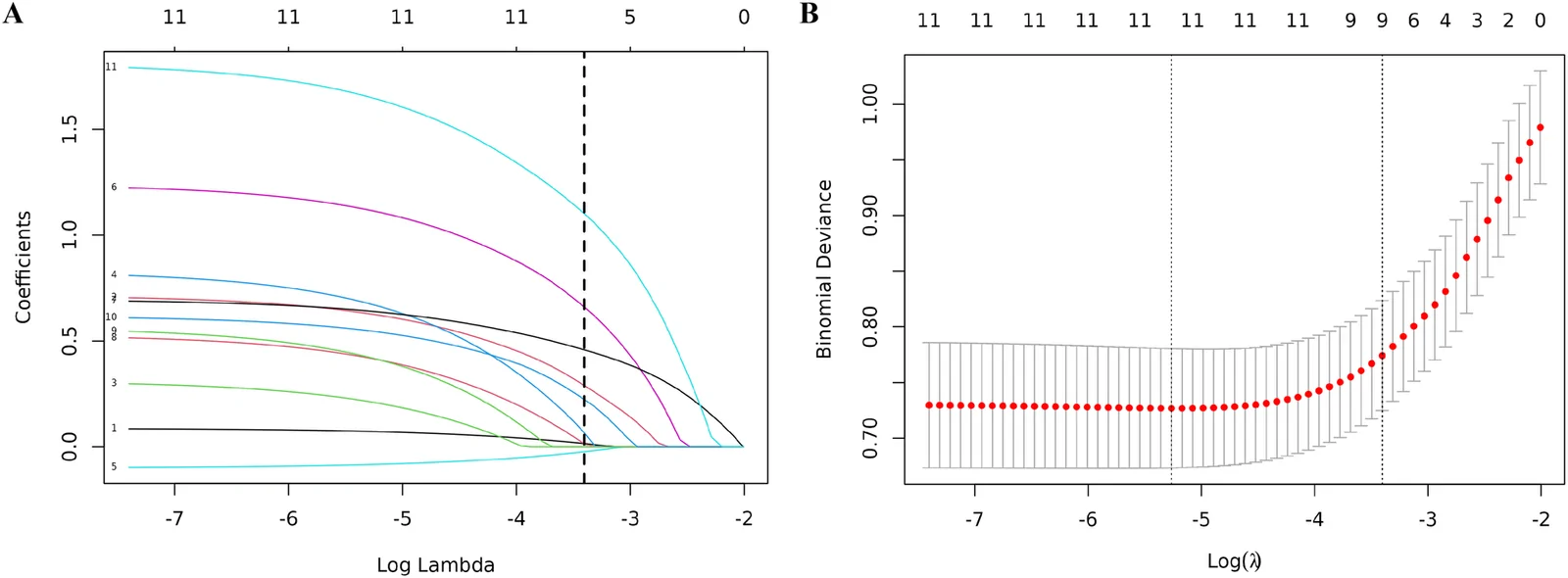
LASSO regression results for variable selection. **(A)** Coefficient path plot; **(B)** Cross-validation error plot. The right dotted line is Lambda at standard error (Lambda.1-SE). The left dotted line is Lambda at minimum error (Lambda.min). Our study selected the variables filtered by Lambda.1-SE, where nine non-zero coefficients were selected (*λ*=0.0333). LASSO, least absolute shrinkage and selection operator; SE, standard error.

The 11 variables chosen using univariate analysis (10-fold cross-validation) were subjected to a LASSO regression analysis. The best *λ* value was chosen using the 1-standard-error rule (*λ*.1se = 0.0333), which selects the most parsimonious model within one standard error of the minimum cross-validation error. Finally, 9 variables with non-zero regression coefficients were retained, namely age, ASA classification, combined COPD, preoperative ALB, preoperative CMMS score, preoperative Frail frailty index, anesthesia method, postoperative hypoxemia, and early postoperative NRS score ≥6 points (Figure 2).

### Multivariable logistic regression study of POD risk variables

The variables chosen using LASSO regression were utilized as independent variables for the multivariate logistic regression analysis, with the dependent variable being whether POD occurred (0 for no incidence, 1 for occurrence). Multivariable logistic regression analysis identified six independent risk factors for POD (Table 3). POD risk was independently influenced by age (OR = 1.092, 95% CI: 1.023–1.166, *P* = 0.008), ASA grade IV (OR = 2.105, 95% CI: 1.131–3.917, *P* = 0.019), preoperative serum ALB level (OR = 0.901, 95% CI: 0.830–0.978, *P* = 0.012), preoperative frailty index (per point increase, OR = 2.021, 95% CI: 1.612–2.534, *P* < 0.001), preoperative CMMS score <27 (OR = 3.608, 95% CI: 1.878–6.931, *P* < 0.001), and early postoperative NRS score ≥6 (OR = 5.998, 95% CI: 3.028–11.880, *P* < 0.001).

**Table 3:** Multivariable logistic regression analysis of risk factors for POD.

Characteristics	*β*	SE	Wald *χ*^2^	*P*	OR	95%CI
Age	0.088	0.034	6.938	0.008	1.092	1.023–1.166
ASA classification	0.744	0.317	5.512	0.019	2.105	1.131–3.917
Preoperative serum ALB	−0.105	0.042	6.250	0.012	0.901	0.830–0.978
Preoperative CMMS score	1.283	0.333	14.844	<0.001	3.608	1.878–6.931
Preoperative Frail frailty index	0.704	0.115	37.163	<0.001	2.021	1.612–2.534
Early postoperative NRS score	1.791	0.349	26.394	<0.001	5.998	3.028–11.880

Variables are coded as follows: early postoperative NRS score (1 = NRS ≥6, 0 = NRS <6); preoperative CMMS score (1 = CMMS <27, 0 = CMMS ≥27); ASA classification grade (1 = grade IV, 0 = grade III). SE, standard error; OR, odds ratio; CI, confidence interval; ALB, albumin; CMMS, Chinese mini mental status; NRS, numerical rating scale.

### Development of a nomogram and ROC analysis

**Figure 3:**
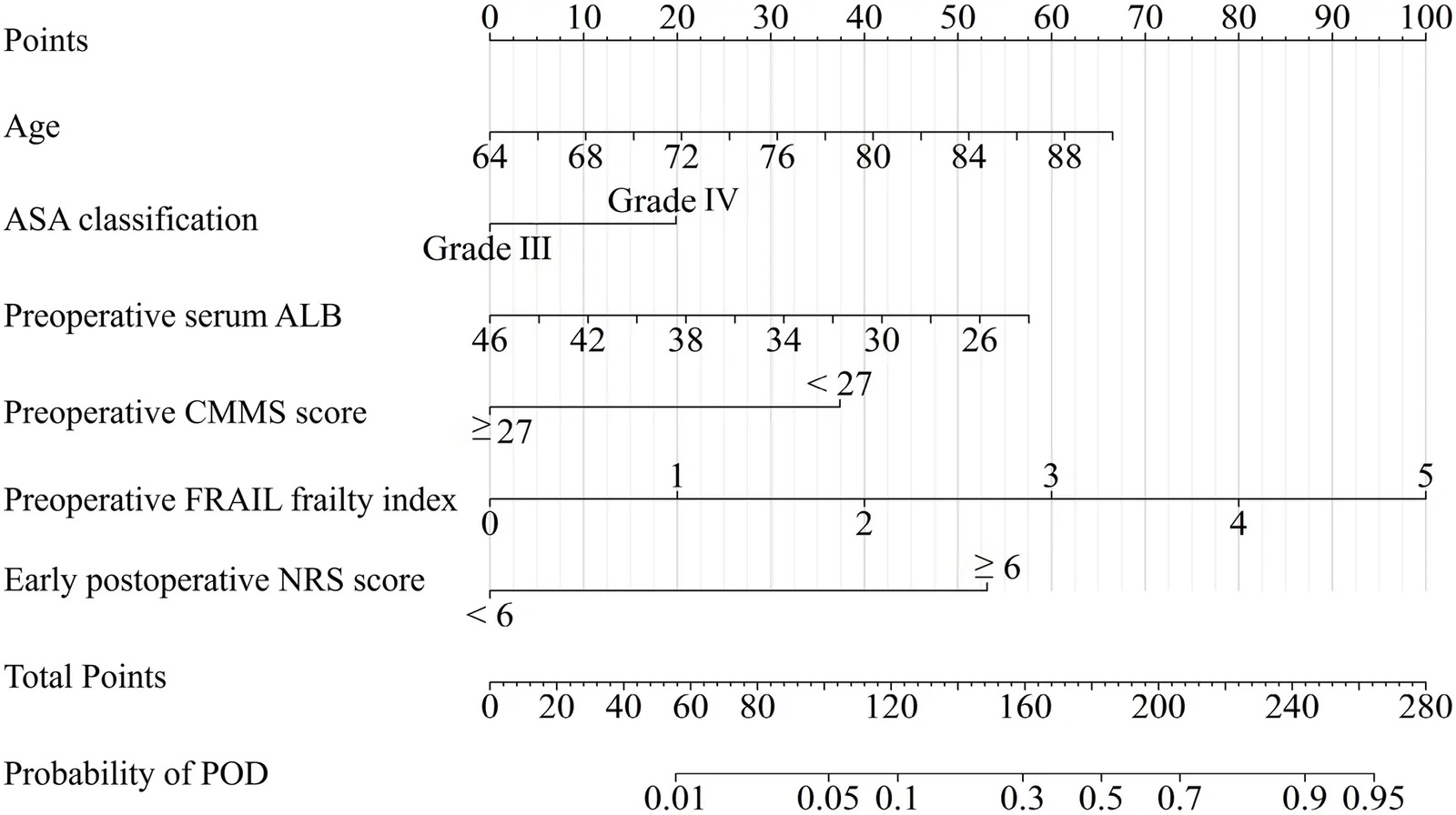
Nomogram for predicting POD in elderly patients. POD, postoperative delirium; ASA, American Society of Anesthesiologists; ALB, albumin; CMMS, Chinese mini mental status; NRS, numerical rating scale.

**Figure 4:**
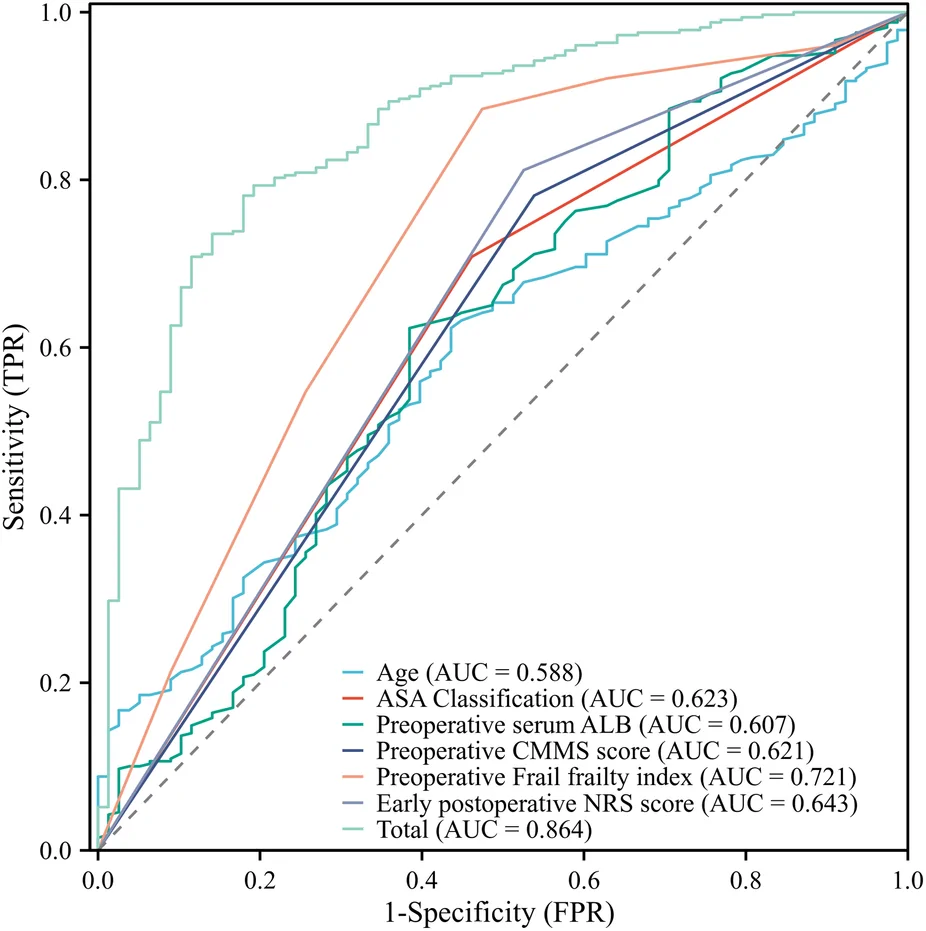
ROC curves of individual predictors and the combined model for predicting POD. ROC, receiver operating characteristic; AUC, area under the curve; POD, Postoperative delirium; ASA, American Society of Anesthesiologists; ALB, albumin; CMMS, Chinese mini mental status; NRS, numerical rating scale.

A nomogram was created to individually estimate the probability of POD in older patients receiving hip or knee arthroplasty based on the six independent factors found using multivariable logistic regression (Figure 3). Figure 4 displays the ROC curves for both the entire model and the individual predictors. At the ideal cut-off value of 0.189, the entire model's AUC was 0.864 (95% CI: 0.819–0.909), with a sensitivity of 78.1% and a specificity of 82.1% (Table 4). As detailed in Table 4, among the individual predictors, the preoperative frailty index exhibited the highest predictive value (AUC=0.721), followed by the early postoperative NRS score (AUC=0.643), ASA classification (AUC=0.623), preoperative CMMS score (AUC=0.615), preoperative serum ALB level (AUC=0.607), and age (AUC=0.588).

**Table 4:** ROC analysis results for predictors of POD.

Characteristics	AUC	Sensitivity	Specificity	Youden index	cut-off value	95%CI
Age	0.588	0.623	0.564	0.187	77.650	0.523–0.653
ASA classification	0.623	0.708	0.538	0.247	0.500	0.562–0.684
Preoperative serum ALB	0.607	0.623	0.615	0.238	33.550	0.533–0.681
Preoperative CMMS score	0.615	0.781	0.449	0.230	0.500	0.555–0.675
Preoperative Frail frailty index	0.721	0.885	0.526	0.410	2.500	0.654–0.788
Early postoperative NRS score	0.643	0.812	0.474	0.286	0.500	0.583–0.703
Total	0.864	0.781	0.821	0.602	0.189	0.819–0.909

ROC, receiver operating characteristic; POD, postoperative delirium; ASA, American Society of Anesthesiologists; ALB, albumin; CMMS, Chinese mini mental status; NRS, numerical rating scale.

### Model validation

**Figure 5:**
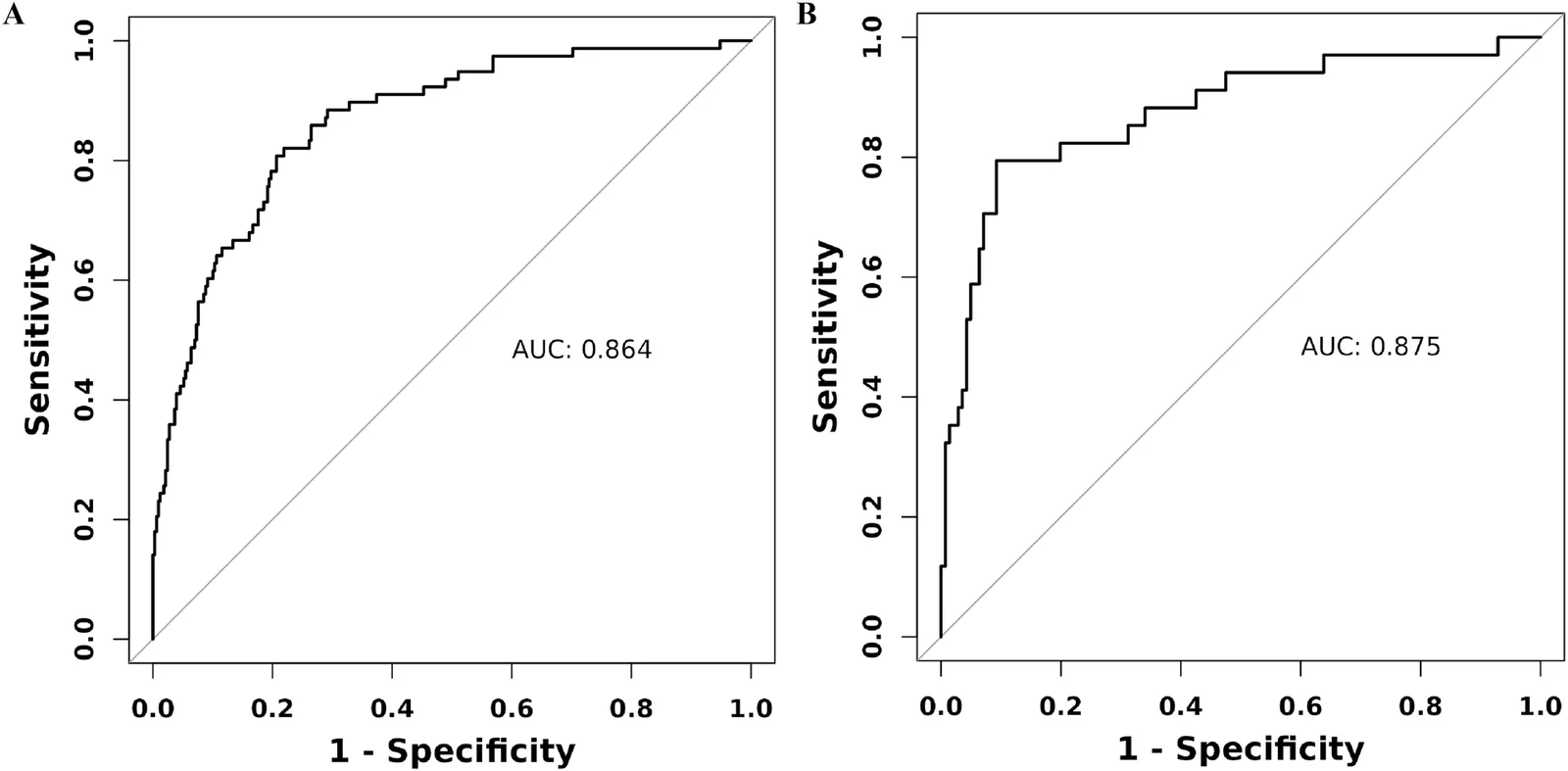
ROC curves of the training cohort and the validation cohort. **(A)** training cohort, **(B)** validation cohort. ROC, receiver operating characteristic; AUC, area under the curve.

**Figure 6:**
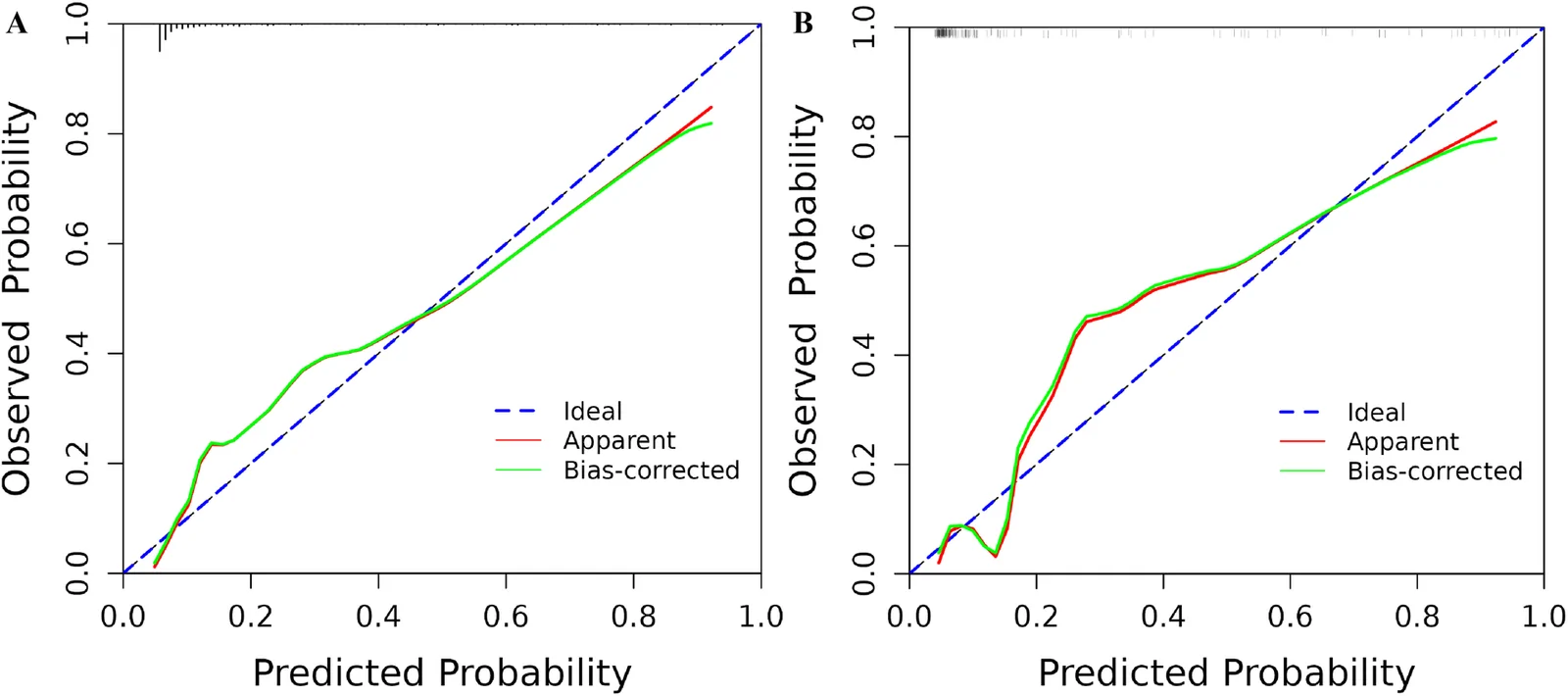
Calibration curves of the training cohort and the validation cohort. **(A)** training cohort, **(B)** validation cohort.

**Figure 7:**
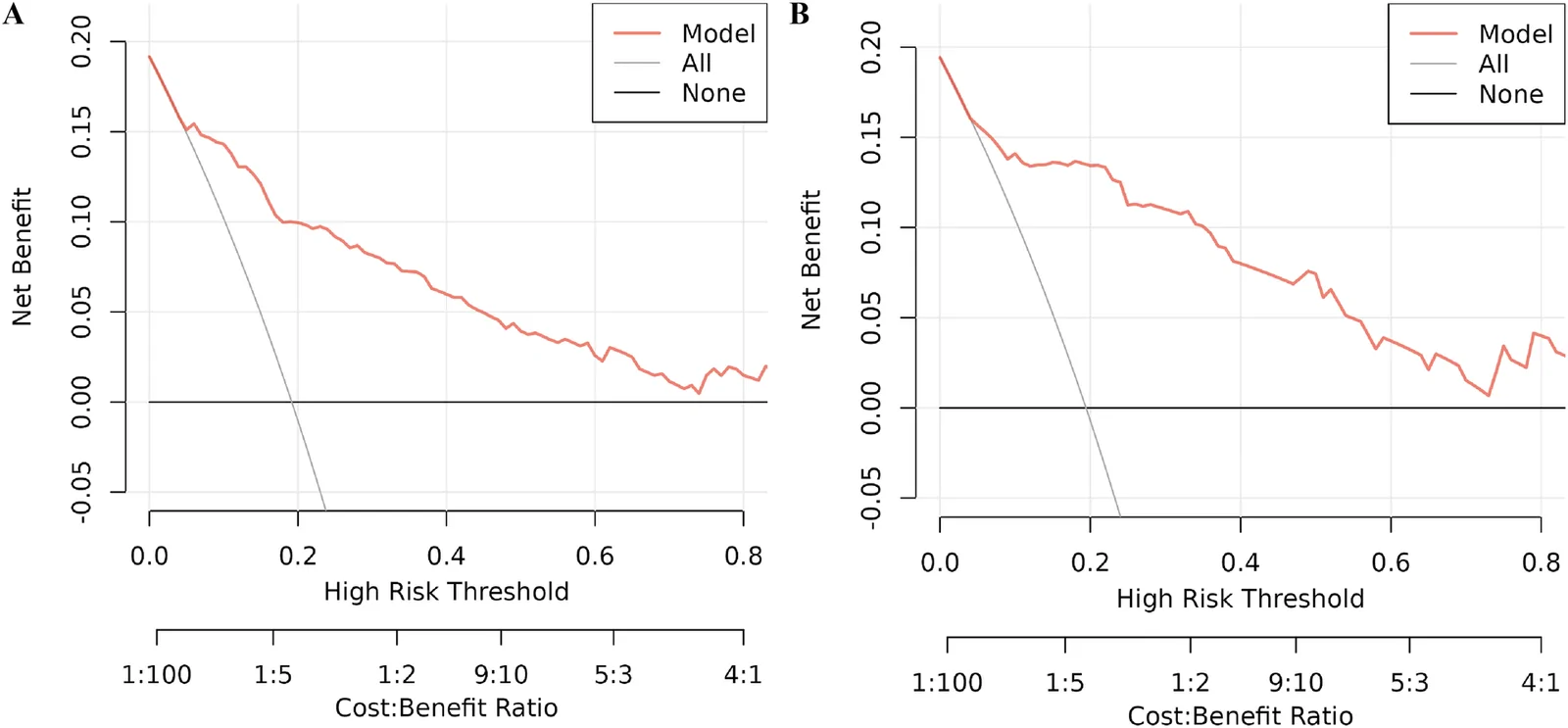
Decision curve analysis of the training cohort and the validation cohort. **(A)** training cohort, **(B)** validation cohort.

The training cohort's AUC was 0.864 (95% CI: 0.819–0.909) (Figure 5A), whereas the validation cohort's was 0.875 (95% CI: 0.800–0.949) (Figure 5B). Figure 6 displays the calibration curves. The apparent calibration curve and the bias-corrected curve in the training cohort (Figure 6A) only slightly deviated from the ideal calibration line in the low-probability range. In the validation cohort (Figure 6B), the bias-corrected curve was also close to the ideal line, while the apparent calibration curve was slightly lower than the ideal line in the medium- to high-probability range, yet still maintained good overall calibration consistency. The training cohort's Brier score was 0.107, whereas the validation cohort's was 0.094, indicating good calibration performance. DCA (Figure 7) showed that the net benefit of the logistic regression model was greater than the “treat-all” and “treat-none” approaches in both the training cohort (Figure 7A) and the validation cohort (Figure 7B) within a broad range of high-risk thresholds. The benefit was especially noticeable in the probability range of 8% to 45%, indicating that this model can assist accurate, customized clinical decision-making and has good application value in clinical practice.

### Results of SHAP interpretability analysis

**Figure 8:**
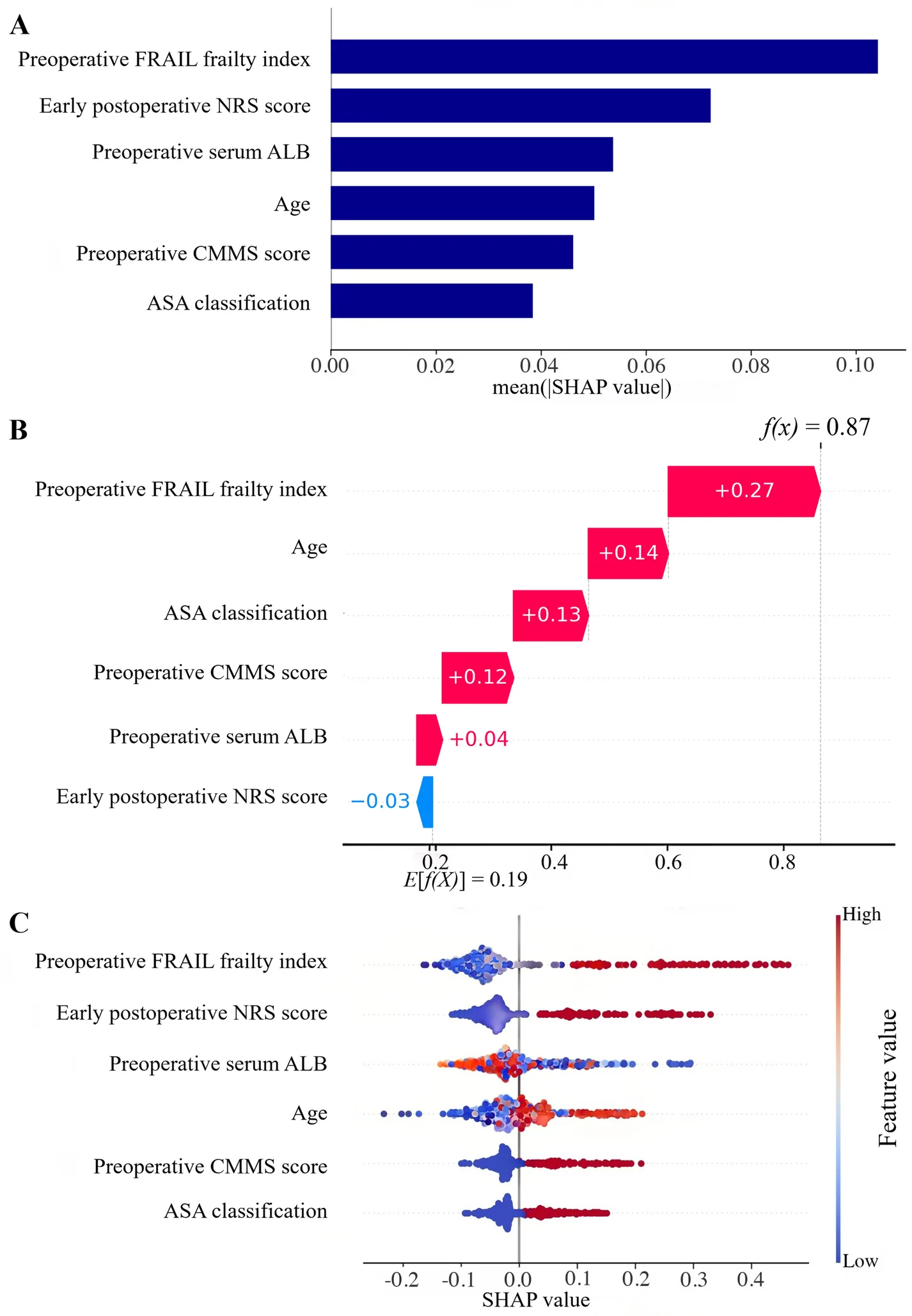
Model interpretability analysis based on SHAP. **(A)** Feature importance bar plot. The plot displays variables ranked by their mean absolute SHAP values. **(B)** Single-sample SHAP waterfall plot. The plot illustrates how the prediction [f**(x)** = 0.87] is decomposed relative to the baseline expected value {E[f(x)] = 0.19}. Red bars indicate positive contributions to POD risk; blue bars indicate negative contributions. The preoperative frailty index was the dominant positive contributor in this case. **(C)** SHAP beeswarm plot. The plot shows the distribution of SHAP values across all samples. The *x*-axis represents SHAP values; the *y*-axis lists features by importance. Color from blue to red reflects feature values from low to high. Higher frailty index, early postoperative NRS score, and age, as well as lower albumin and CMMS scores, were associated with increased POD risk. SHAP, SHapley Additive exPlanations; POD, postoperative delirium; ASA, American Society of Anesthesiologists; ALB, albumin; CMMS, Chinese mini mental status; NRS, numerical rating scale.

We used SHAP analysis to thoroughly evaluate the model behavior in order to gain a better understanding of the prediction model's decision-making process and make clear how each variable influences the final output (Figure 8). The preoperative frailty index was the most significant variable, as indicated by the feature importance plot (Figure 8A), with the greatest mean absolute SHAP value. This suggests that baseline frailty state is a major predictor of postoperative delirium. The pretreatment serum ALB level and the early postoperative NRS score came next in significance. Figure 8B displays the contribution of each feature for a typical high-risk case. Beginning from the baseline expected prediction value, the final predicted probability of POD was notably increased, mainly driven by a high frailty index. Other factors, including older age, higher ASA classification, lower preoperative CMMS score, and relatively low serum ALB, further elevated the model output, collectively pushing the patient into a high-risk category. The overall pattern of feature effects is summarized in the SHAP beeswarm plot (Figure 8C). Positive SHAP values were associated with higher frailty index, early postoperative NRS score, age, and ASA categorization, suggesting a corresponding rise in the anticipated risk of POD. Higher preoperative CMMS scores and serum ALB levels, on the other hand, tended to produce negative SHAP values, which indicate a decreased projected risk. These directional associations are consistent with existing clinical understandings of perioperative risk factors, supporting that the model captures biologically meaningful relationships rather than random statistical patterns.

## Discussion

POD is a frequent central nervous system side effect following hip and knee replacement surgery in older adults. It is strongly linked to long-term cognitive impairment and higher death rates in addition to lengthening hospital stays and adding to medical expenses (28). As a result, early detection and accurate risk prediction are crucial from a therapeutic standpoint. Based on clinical data from 582 elderly patients having hip and knee replacements, this study screened and validated six independent risk factors using multivariable logistic regression and LASSO regression before creating a visual nomogram. In both the training and validation cohorts, the model demonstrated strong discrimination, calibration, and clinical net benefit, offering a useful tool for early risk stratification in clinical practice. SHAP analysis further revealed that the frailty index was the most influential predictor, with the highest mean absolute SHAP value, followed by postoperative NRS score and preoperative ALB.

There are distinct clinical and physiological underpinnings for each of the six independent risk factors found in this study. One of the most well-known risk factors for POD is age. The brain is far more vulnerable to surgical stress and anesthetic medications as we age due to decreased cerebral blood flow, unbalanced neurotransmitter production, and altered blood-brain barrier permeability. Advanced age is one of the most consistently identified risk factors for POD, with multiple studies demonstrating a significant independent association between increasing age and POD risk (6). ASA classification level IV reflects that patients have serious life-threatening systemic diseases. These patients have poor organ reserve function, large physiological fluctuations during the perioperative period, and are more prone to cerebral hypoperfusion and inflammatory responses, thereby inducing delirium (29). The OR value of ASA IV level in this study reached 2.105, suggesting that optimizing the management of comorbidities before surgery is of vital importance.

Preoperative ALB was an independent protective factor for POD (OR = 0.901), meaning that the risk increased with decreasing ALB levels. Hypoalbuminemia is an independent risk factor for postoperative sub-delirium syndrome, according to a recent prospective study on elderly patients with hip fractures (30). ALB contains anti-inflammatory and antioxidant properties in addition to being a nutritional status indicator. Hypoalbuminemia can lead to brain tissue edema, neurotransmitter synthesis disorders, and weaken the body's immune regulatory ability against surgical trauma. Therefore, preoperative nutritional support and correction of hypoalbuminemia may become an effective intervention target for reducing POD. Preoperative cognitive dysfunction (CMMS <27) increased POD risk by 3.6-fold (OR = 3.608). Patients with cognitive decline have reduced brain reserve and fragile neurotransmitter balance, making them more vulnerable to acute brain dysfunction under anesthesia and surgical stress (31). The study's findings highlight the importance of preoperative cognitive assessment once more.

Frailty is the core variable in this study. Frailty is a multisystem decline syndrome in older adults, characterized by reduced physiological reserve and increased stress susceptibility. The FRAIL scale assesses frailty through five dimensions: fatigue, resistance, ambulation, illness, and weight loss (32). According to our research, there is a dose-dependent link between the severity of frailty and the probability of POD. The frailty index, when considered as a continuous variable, produced an OR of 2.021 per point increase. This finding aligns with recent studies demonstrating that preoperative frailty strongly predicts POD in orthopedic surgery patients (33). The mechanisms linking frailty to POD involve chronic low-grade inflammation and neuroinflammation. Frail individuals exhibit elevated baseline levels of pro-inflammatory cytokines, which can be further exacerbated by surgical stress, predisposing them to acute brain dysfunction (34). Notably, sustained NLRP3 expression in the hippocampus has been observed in aging, linking peripheral inflammation to central nervous system vulnerability. Such neuroinflammatory pathways may amplify the impact of surgical stress on brain function, thereby increasing POD susceptibility in frail patients.

SHAP analysis further underscores the dominant role of frailty. Through the quantification of each variable's contribution to individual forecasts, SHAP demonstrated that the frailty index consistently outperformed the influence of other clinical parameters, contributing the largest magnitude to model output with the highest mean absolute SHAP value. This interpretability approach confirms that frailty is not merely a statistical association but the central driver of POD risk in this high-risk population, highlighting its potential as a key target for preoperative optimization. In fragile elderly patients undergoing total joint replacement, preoperative frailty diagnosis and care may be a promising technique to lower POD incidence and enhance postoperative outcomes (35).

Early postoperative moderate-to-severe pain (NRS ≥6, OR = 5.998) was identified as a strong independent risk factor for POD, as it activates the sympathetic nervous system, releases pro-inflammatory cytokines, disrupts sleep rhythm, and increases overall physiological stress, all of which can compromise brain function in vulnerable patients (36). Given that postoperative pain is a controllable factor, optimizing analgesia protocols represents a practical and effective approach to lowering POD risk in this high-risk population.

The complicated regression equation was transformed into an easy-to-use scoring tool by creating a nomogram based on the six criteria that were found. This allowed clinicians to rapidly predict the probability of each patient's POD. The model's discriminative ability was 0.864 in the training set and 0.875 in the validation set, significantly outperforming any single risk factor, demonstrating the advantage of multifactor combined prediction. Calibration curves and decision curve analysis further confirmed its accuracy and clinical utility. Compared with previous studies, our model incorporates frailty as a continuous measure and is specifically tailored to the Chinese elderly hip and knee arthroplasty population, enhancing its relevance and applicability (37).

There are several restrictions on this study. First, selection bias may have been introduced because the sample for this single-center retrospective study came from a single institution without external validation. Additionally, the long study period may have been affected by evolving perioperative management strategies. Second, although the model included multiple clinical indicators, it did not incorporate biological markers such as genetic or inflammatory factors, nor did it comprehensively assess potential confounders like postoperative sedative use or sleep quality, leaving room for further improvement in predictive performance. Future multicenter prospective research is necessary to confirm the generalizability of the paradigm. Integrating omics data to discover novel biomarkers and developing dynamic prediction systems based on real-time physiological parameters could further refine risk stratification and support individualized POD prevention.

## Conclusion

A nomogram for predicting POD risk in older patients following hip or knee replacement was successfully created and validated in this study. The model incorporates six readily available clinical factors: age, ASA grade IV, preoperative serum albumin, preoperative CMMS score <27, preoperative frailty index, and early postoperative NRS score ≥6. It demonstrates good discrimination, calibration, and clinical net benefit, providing a simple and intuitive tool for early identification of high-risk patients. This nomogram can help physicians apply focused preventative measures, which may lower the incidence of POD and enhance the quality of life and postoperative results for older patients.

## Data Availability

The original contributions presented in the study are included in the article/Supplementary Material, further inquiries can be directed to the corresponding author.
